# Inhibition of allergic airway responses by heparin derived oligosaccharides: identification of a tetrasaccharide sequence

**DOI:** 10.1186/1465-9921-13-6

**Published:** 2012-01-23

**Authors:** Tahir Ahmed, Gregory Smith, Iontcho Vlahov, William M Abraham

**Affiliations:** 1 Department of Research, Mount Sinai Medical Center, Miami Beach, Florida, USA; 2 Endocyte Inc. West Lafayette, IN, USA

## Abstract

**Background:**

Previous studies showed that heparin's anti-allergic activity is molecular weight dependent and resides in oligosaccharide fractions of <2500 daltons.

**Objective:**

To investigate the structural sequence of heparin's anti-allergic domain, we used nitrous acid depolymerization of porcine heparin to prepare an oligosaccharide, and then fractionated it into disaccharide, tetrasaccharide, hexasaccharide, and octasaccharide fractions. The anti-allergic activity of each oligosaccharide fraction was tested in allergic sheep.

**Methods:**

Allergic sheep without (acute responder) and with late airway responses (LAR; dual responder) were challenged with *Ascaris suum *antigen with and without inhaled oligosaccharide pretreatment and the effects on specific lung resistance and airway hyperresponsiveness (AHR) to carbachol determined. Additional inflammatory cell recruitment studies were performed in immunized ovalbumin-challenged BALB/C mice with and without treatment.

**Results:**

The inhaled tetrasaccharide fraction was the minimal effective chain length to show anti-allergic activity. This fraction showed activity in both groups of sheep; it was also effective in inhibiting LAR and AHR, when administered after the antigen challenge. Tetrasaccharide failed to modify the bronchoconstrictor responses to airway smooth muscle agonists (histamine, carbachol and LTD_4_), and had no effect on antigen-induced histamine release in bronchoalveolar lavage fluid in sheep. In mice, inhaled tetrasaccharide also attenuated the ovalbumin-induced peribronchial inflammatory response and eosinophil influx in the bronchoalveolar lavage fluid. Chemical analysis identified the active structure to be a pentasulfated tetrasaccharide ([IdoU2S (1→4)GlcNS6S (1→4) IdoU2S (1→4) AMan-6S]) which lacked anti-coagulant activity.

**Conclusions:**

These results demonstrate that heparin tetrasaccharide possesses potent anti-allergic and anti-inflammatory properties, and that the domains responsible for anti-allergic and anti-coagulant activity are distinctly different.

## Introduction

Heparin is a highly sulfated, linear polysaccharide that has multiple biological activities [1-3]. Heparin inhibits blood coagulation [1], but also has numerous "non-anticoagulant" functions, including interaction with various growth factors [4,5], modulation of cellular proliferation [6,7] and regulation of angiogenesis [8]. Heparin also modulates various proteases and enzymes [9-11] and possesses anti-inflammatory and immunoregulatory activities [12-14]. Thus, inhaled heparin has been shown to inhibit allergic airway responses in sheep [15], as well as to prevent the bronchoconstrictor responses to exercise and antigen in asthmatic subjects [16-19]. Many biological actions of heparin, including the anticoagulant and the anti-allergic activity are molecular weight dependent [20-22]. In allergic sheep, an inverse relationship between molecular weight and the anti-allergic activity of fractionated heparin was observed, with ultralow molecular weight heparin found to be the most potent fraction [21-23].

The basic polymeric structure of glycosaminoglycan heparin is an alternating sequence of disaccharide units comprising of repeating 1→4 linked L-iduronic acid and D-glucosamine residues [2,3]. The sugar sequence, degree of sulfation and its high charge density are the basis of heterogenous molecular organization of heparin and its ability to interact with various proteins causing their activation, deactivation, or stabilization [2,3,24]. Heparin's structural heterogeneity is linked to its multiplicity of actions. For example, the binding domain to antithrombin III [25], and basic fibroblast growth factor [4] demonstrate the relationship between the fine structure of heparin derived oligosaccharides and biological functions. The antithrombin III binding site requires a minimal pentasaccharide sequence [25], while the binding domain to basic fibroblast growth factor requires a hexasaccharide sequence [4].

Consistent with these observations, our previous studies have demonstrated that the anti-allergic activity of heparin is independent of its anti-coagulant properties and resides in oligosaccharide fractions (<2500 daltons) [23]. However, the exact structural sequence is not known. Therefore, the purpose of this study was to identify the minimal chain length and structural sequence of the anti-allergic domain of heparin. To do this, we prepared an oligosaccharide mixture, used size-exclusion chromatography to obtain disaccharide, tetrasaccharide, hexasaccharide and octasaccharide fractions, and then determined their anti-allergic activity.

## Methods

### Ovine Studies

#### Animal Preparation

All procedures used in this study were approved by the Mount Sinai Animal Research Committee, which is responsible for ensuring the humane care and use of experimental animals. Twenty unsedated adult female sheep, with an average weight of 31 kg (27-36 kg), were suspended in an upright position in a specialized body harness in a modified shopping cart, with their heads secured as published previously [26,15]. All sheep were allergic to *Ascaris *suum antigen and had previously been shown to develop bronchoconstriction following inhalation challenge with the antigen, either without (acute responders) or with late airway responses (dual responders) [27].

#### Airway Mechanics

Measurements of mean pulmonary airflow resistance, in units of cmH_2_O/L/s, and thoracic gas volume, in liters, were made by the esophageal balloon technique and body plethysmography, respectively, as previously described [27,15]. Data were expressed as specific lung resistance (SR_L _= mean pulmonary airflow resistance × thoracic gas volume) in cmH_2_O/sec.

#### Aerosol Delivery System

All aerosols were generated using a disposable medical nebulizer (Raindrop, Puritan Bennett, Lenexa, KS). The nebulizer was connected to a dosimeter system consisting of a solenoid valve and a source of compressed air. The output of the nebulizer was directed into a plastic t-piece, which was interconnected between the inspiratory port of the Harvard animal respirator and the endotracheal tube. The solenoid valve was activated for 1 sec at the beginning of the inspiratory cycle of the respirator. Aerosols were delivered at a tidal volume of 500 ml and a rate of 20 breaths/minute. Various heparin oligosaccharide fractions were dissolved in 3 ml of bacteriostatic injection water and administered as an aerosol over 15-20 minutes. Aerosols of *Ascaris *suum extract (diluted 20:1 with phosphate buffered saline; 82000 protein nitrogen units/ml) and carbachol were also generated with this nebulizer system.

#### Bronchial Reactivity to Carbachol

To assess baseline airway responsiveness, cumulative dose-response curves to inhaled carbachol were performed on experiment day 1 by measuring SR_L_, before and immediately after inhalation of buffered saline and after each administration of 10 breaths of increasing concentrations of carbachol (0.25%, 0.5%, 2.0%, 3.0% and 4.0% wt/vol solution). The bronchoprovocation was discontinued when SR_L _increased to 400% above the baseline. The cumulative provocating dose of carbachol (in breath units) that increased SR_L _to 400% above the baseline was calculated (PD_400_). One breath unit was defined as one breath of 1% carbachol solution. Baseline dose-response curves to carbachol were performed in all sheep at least 2 weeks after the last exposure to antigen [27,15,28].

#### Bronchoalveolar Lavage (BAL)

The distal tip of a specifically designed 80 cm fiberoptic bronchoscope was wedged into a randomly selected subsegmental bronchus. BAL was performed by an infusion and gentle aspiration of 30 ml aliquots of phosphate buffered saline (pH 7.4) at 39°C, using 30 ml syringes attached to the working channel of the bronchoscope. The effluent was filtered through a single layer of gauze and placed immediately on ice. The volume of the effluent collected from the BAL fluid was measured and centrifuged at 420 g, at 4°C for 15 minutes. The supernatant was decanted and centrifuged again at 1000 g, at 4°C for 15 minutes. The supernatant was frozen at -80°C for subsequent histamine analysis.

#### Histamine Radioimmunoassay

Duplicate aliquots from each BAL sample were used for histamine radioimmunoassay using a commercial kit from Immunotech International (AMAC Inc; Westerbrook, ME). The sensitivity of the assay is 0.05-2.0 nM, and coefficient of variation is <10%. There is less than 0.1% cross-reactivity with histidine, serotonin or t-methyl histamine.

### Murine Studies

These studies were also approved by the Mount Sinai Animal Research Committee. Female BALB/c mice, 4-6 weeks old were immunized by injecting intra-peritoneally with 0.2 ml of 0.05% solution of ovalbumin adsorbed to alum on day 1 and again on day 14. On days 25, 26, & 27, the animals were placed in an exposure chamber for ovalbumin aerosol challenge (3% ovalbumin for 30 minutes). Aerosols were generated using a PARi IS-2 nebulizer at a flow rate of 6 l/min. 24 hours later, on day 28, the mice were sacrificed, the trachea was carefully dissected and a blunt needle was inserted. Bronchoalveolar lavage (BAL) was performed with freshly prepared phosphate buffered saline (PBS, 0.8 ml) at 4°C. The BAL fluid was centrifuged at 1500 RPM for 10 minutes. The supernatant was separated and the pellet was resuspended in PBS to a volume of 0.3 ml, and centrifuged again at 650 RPM for 10 minutes. The slides were prepared and stained with a Giemsa-Wright stain. In some mice, after the BAL was completed, the lungs were dissected, fixed in formalin and embedded in paraffin for histology. The tissues were cut into 3 um sections and stained with hematoxylin and eosin stain.

### Preparation of Heparin Oligosaccharides

An aqueous solution of porcine intestinal heparin (USP) was depolymerized at room temperature with HNO_2_, generated *in situ *from HCl and NaNO_2 _(pH: 1.5). After one hour, the reaction pH was raised to 10 by addition of aqueous NaOH. The solution was then reacted with NaBH_4 _for 18 hours to reduce the aldehyde group in the terminal 2,5-anhydro-D-mannose, obtained in the initial depolymerization step [29]. Reaction mixture was then neutralized and subjected to freeze-drying, which yielded the sodium salt of oligosaccharide mixture. Size exclusion gel chromatography of the oligosaccharide mixture was performed on 1.5 M × 80 cm column, containing BioRad P4 Biogel (10 L) and eluting with 0.2 M NH_4_HCO_3_. After lyophilization, an ammonium salt of the appropriate fractions was obtained. The salt form was exchanged to sodium by passing the aqueous solution of the ammonium salt of each fraction through a column containing Amberlite 1R 120 PLUS cation-exchange Resin (Sigma Aldrich Chemical Co.). Finally, after freeze-drying the sodium salt of each oligosaccharide fraction (i.e. disaccharide, tetrasaccharide, hexasaccharide and octasaccharide) was collected.

### Characterization of Heparin Oligosaccharides

The size of the sodium salt of each heparin-derived oligosaccharide fraction, including disaccharide, tetrasaccharide, hexasaccharide and octasaccharide was determined by size exclusion HPLC [30]. Further structural confirmation of the disaccharide, tetrasaccharide, hexasaccharide fractions was obtained by ^1^H-NMR, ^13^C-NMR, and Mass Spectrometry [30-32].

### Experimental Protocol

Ovine Studies:

#### A. Effect of Heparin Oligosaccharides in Acute Responders

For the control experiment, after the baseline measurements of SR_L_, the sheep (n = 6) were challenged with aerosolized antigen and measurements of SR_L _were repeated within five minutes post-challenge. To evaluate the effect of heparin oligosaccharides on this antigen-induced response, dose-response studies were performed. The inhaled doses of various oligosaccharides used were: (a) oligosaccharide mixture (n = 6) 0.25 mg/kg, 0.5 mg/kg and 1 mg/kg; (b) octasaccharide (n = 5), 0.125 mg/kg, 0.25 mg/kg, and 0.5 mg/kg; (c) hexasaccharide (n = 5), 0.03 mg/kg, 0.06 mg/kg and 0.125 mg/kg; (d) tetrasaccharide (n = 5), 0.03 mg/kg, 0.06 mg/kg and 0.125 mg/kg; (e) disaccharide (n = 3) was evaluated only at a dose of 4 mg/kg. The sheep were pretreated with different doses of oligosaccharides, 30 minutes before the antigen challenge and measurements of SR_L _were repeated within five minutes post antigen challenge. The interval between tests with each dose of the different oligosaccharide fractions was at least two weeks.

#### B. Effect of Heparin Oligosaccharides in Dual Responders (n = 6)

For every oligosaccharide tested, each sheep was studied on three different experiment days. For the control experiments, baseline bronchial reactivity to carbachol (PD_400_) was determined on experiment day 1. On experiment day 2, after the baseline measurements of SR_L_, each animal was challenged with aerosolized *Ascaris suum *antigen. Repeat measurements of SR_L _were obtained within five minutes after the antigen challenge, and serially for up to eight hours for demonstration of early airway response (EAR) and late airway response (LAR). Twenty-four hours after the antigen challenge, bronchial reactivity to carbachol was re-determined (experiment day 3) as an index of antigen-induced airway hyperresponsiveness (AHR).

The above-mentioned 3-day protocol was repeated, at least two weeks apart for each dose of the oligosaccharide tested. In each sheep, the baseline PD_400 _of carbachol was determined on experiment day 1, followed by experiment day 2, when the sheep were pretreated with the different doses of the inhaled oligosaccharides, 30 minutes before the antigen challenge and then on experiment day 3, the PD_400 _was re-determined. The doses of oligosaccharides used were: (a) oligosaccharide mixture 0.5 mg/kg; (b) octasaccharide, 0.5 mg/kg; (c) hexasaccharide, 0.125 mg/kg; (d) tetrasaccharide, 0.03 mg/kg, 0.06 mg/kg and 0.125 mg/kg.

##### Post-Antigen Administration of Tetrasaccharide (n = 5)

To evaluate the effect of "post-antigen" administration of tetrasaccharide on LAR and AHR, the 3-day protocol described above was repeated, except that instead of pre-treating the animals, the doses of tetrasaccharide (0.03 mg/kg; 0.06 mg/kg; 0.125 mg/kg) were administered immediately after the post-antigen measurements of SR_L_.

#### C. Agonist-Induced Bronchoconstriction (n = 3)

After obtaining the baseline measurements of SR_L_, the sheep were challenged with aerosolized carbachol (10 breaths of 2% solution; n = 3) histamine (50 breaths of 5% solution; n = 3) or leukotriene (LT)D_4 _(20 breaths of 0.01% solution; n = 3) and measurements of SR_L _were repeated immediately after. On separate days, the sheep were pretreated with inhaled tetrasaccharide (0.125 mg/kg) 30 minutes before agonist challenge and then the above protocols were repeated.

#### D. Histamine Release in BAL (n = 6)

In each animal BAL was performed on two different experiment days, at least two weeks apart, before and after a segmental antigen-challenge, without and after pretreatment with inhaled tetrasaccharide (0.125 mg/kg). Tetrasaccharide was nebulized 30 minutes before the segmental antigen challenge. *Ascaris suum *antigen (2.5 ml *Ascaris suum *and 2.5 ml buffer) was infused via a wedged bronchoscope and BAL was performed 20 minutes later. The BAL effluent was centrifuged and the supernatant saved and frozen at -80°C for subsequent RIA.

### Murine Studies

The mice were immunized with intraperitoneal injection of ovalbumin (0.2 ml of 0.05% solution) on day 1 and again on day 14. On days 25, 26 and 27, the negative control group (n = 4) received an aerosol of normal saline, while the positive control group (n = 5) was challenged with an aerosol of 3% ovalbumin for 30 minutes. On day 28, the mice were sacrificed and BAL and lung tissue were prepared for estimation of eosinophil influx and airway inflammation. For the treatment group (n = 4), the mice were pretreated with aerosolized tetrasaccharide (0.3% solution) on days 25, 26, and 27, one hour before ovalbumin challenge, and the above mentioned day 28 protocol was repeated. Histological specimens were obtained from one mouse in each group for representative purposes only.

### Statistical Analysis

The data were expressed as mean ± SE. The SR_L _data were analyzed by a two-way analysis of variance with repeated measures, followed by Newman-Keuls pairwise comparison. Area Under the Curve for EAR AUC 0-4_h _and LAR AUC 4-8_h _were calculated using the trapezoid rule for the treatment and control arms in the same animals and compared using a paired t-test. The baseline values of PD_400 _were compared to post-antigen PD_400 _(without and with oligosaccharide) by Friedman's two-way Analysis of Variance followed by non-parametric multiple comparison. Histamine concentration in the sheep BAL was analyzed by a paired t-test. Mouse BAL celluar analysis was first analyzed with a one way analysis of variance followed by Student-Newman-Keuls method to detect pairwise differences. Significance was accepted at P < 0.05.

## Results

### Effect of Heparin Oligosaccharides in Acute Responders

**Figure 1:**
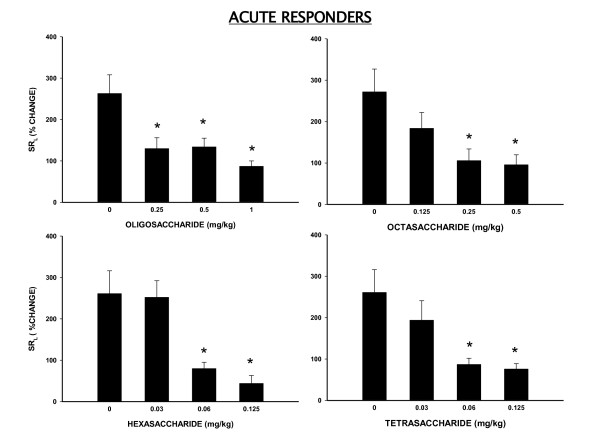
**Effect of inhaled heparin-oligosaccharides on antigen-induced bronchoconstrictor response in "Acute Responder" sheep**. Data are shown as % change in specific lung resistance (SR_L_) ± SE without and after pretreatment with different dose of oligosaccharide mixture (n = 6) and various fractions including octasaccharide (n = 5), hexasaccharide (n = 5) and tetrasaccharide(n = 5). *Significantly different from antigen control (P < .05).

In acute responders (n = 6), mean ± SE peak SR_L _increased by 263 ± 45% with antigen alone. We first tested the oligosaccharide mixture to insure that it had anti-allergic activity similar to what we had seen previously with ultra low molecular weight heparins [21,22]. The inhaled oligosaccharide mixture (MW:1930 daltons) significantly attenuated the peak antigen-induced bronchoconstrictor response (ABR) by 51 ± 10%, 49 ± 9% and 67 ± 7% at doses of 0.25 mg/kg, 0.5 mg/kg and 1 mg/kg, respectively (Figure 1).

Dose response studies with various fractions, isolated from the heparin-oligosaccharide mixture, showed that octasaccharide was the least potent compared to hexasaccharide and tetrasaccharide which were equipotent at the doses tested (Figure 1). The octasaccharide fraction (n = 5, MW:2480 daltons) attenuated the ABR by 32 ± 8% (P = NS), 61 ± 11% (P < .05), and 65 ± 9% (P < .05), at doses of 0.125 mg, 0.25 mg/kg and 0.5 mg/kg, respectively. The hexasaccharide fraction (n = 5, MW: 1890 daltons) attenuated the ABR by 3 ± 6% (P = NS), 69 ± 10% (P < .05), and 83 ± 7% (P < .05) at doses of 0.03 mg/kg, 0.06 mg/kg and 0.125 mg/kg, respectively. Similarly, the tetrasaccharide fraction (n = 5, MW: 1231 daltons) attenuated the ABR by 26 ± 8% (P = NS), 67 ± 7% (P < .05) and 71 ± 7% (P < .05) at doses of 0.03 mg/kg, 0.06 mg/kg and 0.125 mg/kg, respectively. Based on these findings, we considered the 0.5 mg/kg, 0.125 mg/kg and 0.125 mg/kg doses of octasaccharide, hexasaccharide and tetrasaccharide fractions, respectively to be most effective against ABR., At these doses, the octasaccharide fraction was two-fold more potent, and the tetrasaccharide and hexasaccharide fractions were eight-fold more potent than the oligosaccharide mixture.

### Effect of Heparin Oligosaccharides in Dual Responders

**Figure 2:**
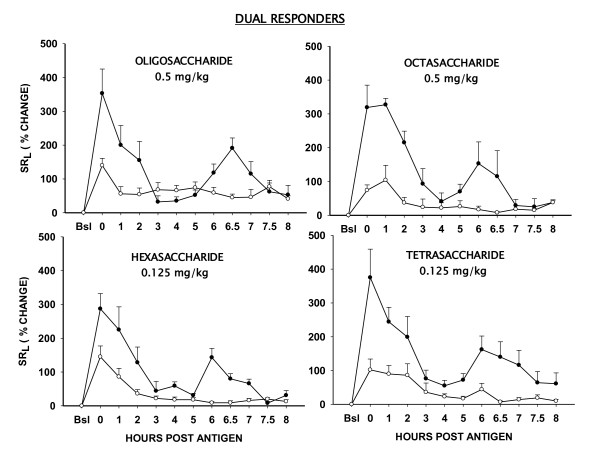
**Effect of inhaled heparin oligosaccharides on antigen-induced early and late airway responses (EAR and LAR) in "Dual Responder" sheep**. Data are shown as % change in specific lung resistance (SR_L_) ± SE without (solid circles) and after (open circles) pretreatment with oligosaccharide mixture (n = 6; 0.5 mg/kg), and various fractions including octasaccharide (n = 3; 0.5 mg/kg), hexasaccharide(n = 5; 0.125 mg/kg), and tetrasaccharide (n = 5; 0.125 mg/kg). Bsl = Baseline.

**Figure 3:**
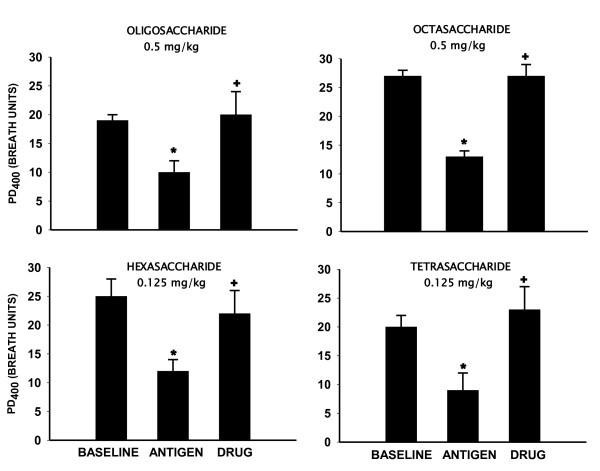
**Effect of inhaled heparin oligosaccharides on antigen-induced airway hyperresponsiveness (AHR) in "Dual Responder" sheep**. Data are shown as PD_400 _(cumulative provocating dose of carbachol in breath units which increased SR_L _by 400%) ± SE for the baseline, and 24 hours post-antigen, without and after pretreatment with oligosaccharide mixture (0.5 mg/kg) and various fractions including Octasaccharide (0.5 mg/kg), hexasaccharide (0.125 mg/kg) and tetrasaccharide (0.125 mg/kg). * Significantly different from baseline (P < .05). + Significantly different from antigen control (P < .05).

Having achieved significant inhibition of the ABR in acute responders, we next tested the activity of the oligosaccharides doses identified above in "Dual Responders''. In this group (n = 6), antigen-induced changes in SR_L _without and after pretreatment with the inhaled heparin oligosaccharides are shown in figure 2. With antigen alone, peak early and late increases in SR_L _were 353 ± 74% and 190 ± 30%, respectively. The corresponding early response area under the curve (EAR AUC_0-4h_) and late response area under the curve (LAR AUC_4-8h_) were 577 ± 132% and 353 ± 73%, respectively. Pretreatment with the inhaled oligosaccharide mixture (0.5 mg/kg) inhibited the antigen-induced increases in SR_L_. Peak early and peak late increases in SR_L _with pretreatment were 140 ± 21% and 77 ± 19%, respectively (P < .05). This translated to an inhibition of EAR AUC_0-4h _and LAR AUC_4-8h _by 60 ± 15%and 52 ± 8%, respectively (P < .05, Figure 2). Heparin-derived oligosaccharide mixture also inhibited the antigen-induced AHR. In the control trial, the 24 hours post-antigen, mean ± SE PD_400 _decreased from 19 ± 1 breath units to 10 ± 2 breath units; this was prevented by the heparin oligosaccharide mixture (PD_400 _= 20 ± 4 breath units). (P < .05, Figure 3).

**Figure 4:**
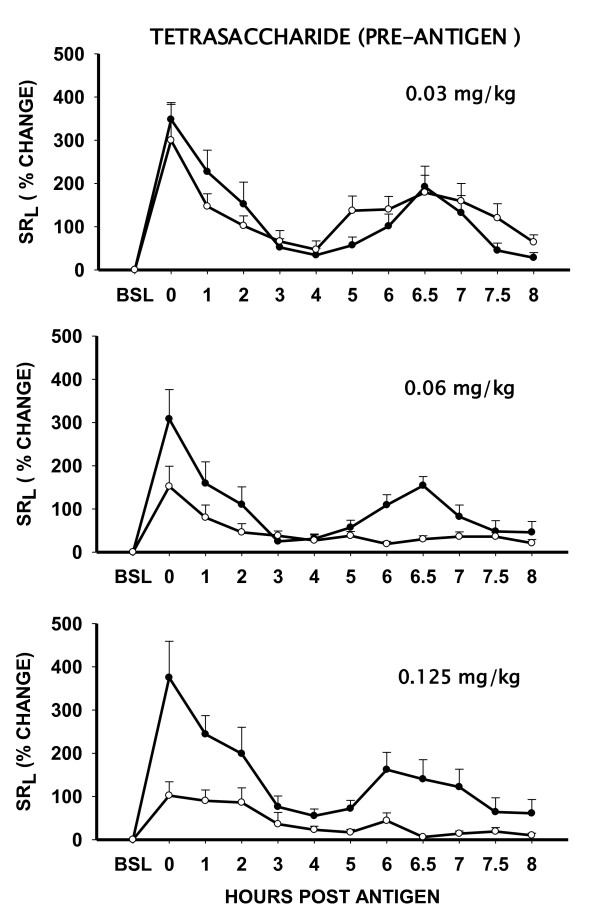
**Dose response effect of pretreatment with inhaled heparin tetrasaccharide on antigen-induced early and late airway responses (EAR and LAR) in "Dual Responder" sheep**. Data are shown as antigen-induced % change in specific lung resistance (SR_L_) ± SE without (solid circles) and after pretreatment (open circles) with inhaled tetrasaccharide (0.03 mg/kg, n = 5); 0.06 mg/kg, n = 6); and 0.125 mg/kg, n = 5). BSL = Baseline.

The octasaccharide fraction of heparin (n = 3; 0.5 mg/kg) inhibited the EAR AUC_0-4h _by 74 ± 10% and LAR AUC_4-8h _by 75 ± 2% (P < .05); while the antigen-induced AHR was inhibited by 100 ± 7%, (P < .05). The hexasaccharide fraction (n = 5; 0.125 mg/kg) inhibited the EAR AUC_0-4h _by 61 ± 8% and LAR AUC_4-8h _by 81 ± 6% (P < .05); while antigen-induced AHR was inhibited by 80 ± 13% (P < .05); respectively. The anti-allergic activity of the tetrasaccharide fraction (n = 5; 0.125 mg/kg) was comparable to the hexasaccharide fraction (Figure 2, 3). Figure 4 shows that the anti-allergic activity of the tetrasaccharide fraction was dose-dependent. While 0.03 mg/kg (n = 5) dose was ineffective, the 0.06 mg/kg (n = 6) and 0.125 mg/kg (n = 5) doses inhibited the EAR AUC_0-4h _by 47 ± 13% and 63 ± 11% (P < .05); LAR AUC_4-8h _by 63 ± 7% and 81 ± 9% (P < .05). Antigen-induced AHR at these doses was inhibited by 95 ± 9% and 100 ± 10% (P < .05); respectively.

#### Post-antigen administration of Tetrasaccharides

**Figure 5:**
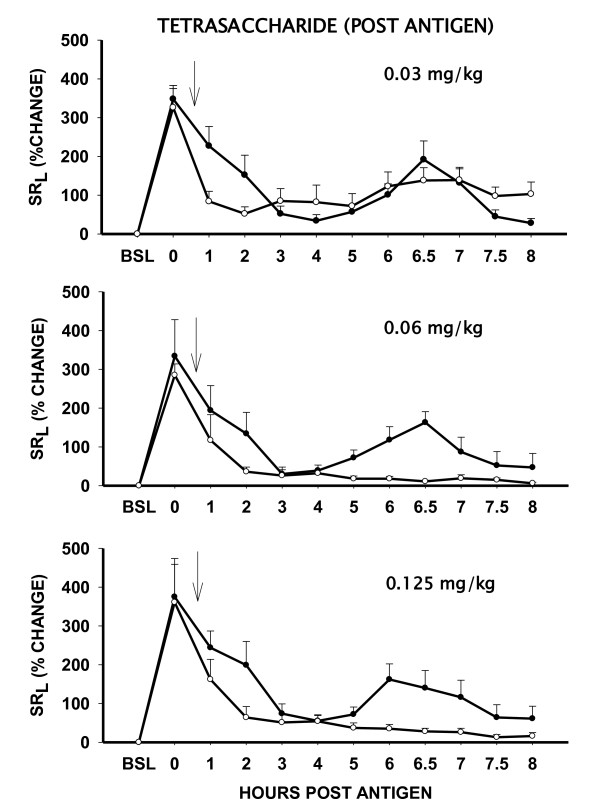
**Dose response effect of inhaled heparin tetrasaccharide, administered "AFTER" the antigen challenge, on antigen-induced LAR**. Data are shown as % change in specific lung resistance (SR_L_) ± SE without (solid circle) and after treatment (open circle) with various doses of tetrasaccharide (n = 5). Tetrasaccharide was administered immediately after the post-antigen measurement of SR_L _(Arrow). BSL = Baseline

Inhaled tetrasaccharide (n = 5) caused a dose-dependent inhibition of LAR AUC_4-8h _and AHR when administered "after" the antigen challenge (Figure 5). While 0.03 mg/kg dose was ineffective, post-antigen administration of 0.06 mg/kg tetrasaccharide inhibited the LAR AUC_4-8h _and AHR by 80 ± 9% and 100 ± 16% (P < .05), respectively. The effects of 0.06 mg/kg and 0.125 mg/kg doses of tetrasaccharide were comparable.

#### Minimal effective chain length

**Figure 6:**
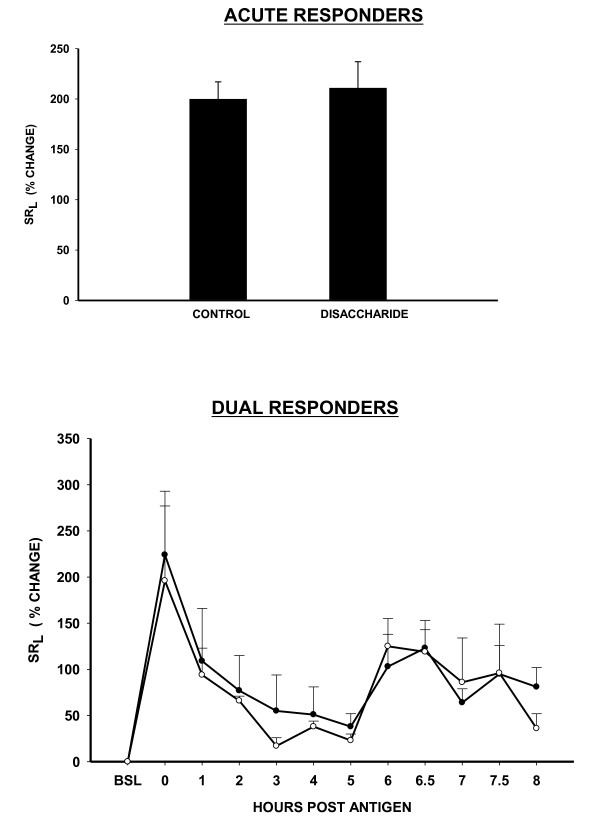
**Failure of inhaled heparin disaccharide to inhibit allergic bronchoconstrictor responses in sheep**. Top: Antigen-induced % change in specific lung resistance (SR_L_) ± SE in "Acute Responders" with (4 mg/kg; n = 3) and without pretreatment (control). Bottom: "Dual Responders" (1 mg/kg, n = 3,) without (control; open circles) and after treatment (solid circles) with inhaled disaccharide.

Heparin derived tetrasaccharide was the minimum effective chain length possessing the anti-allergic activity. The disaccharide fraction was ineffective in both "acute" and "dual" responder sheep. Inhaled disaccharide at a 32-fold higher dosage than tetrasaccharide had no effect on antigen-induced ABR in "acute responders" (Figure 6, Top). Similarly an 8-fold excess of disaccharide had no effect in dual responders (Figure 6, Bottom).

#### Agonist-induced bronchoconstriction

The inhaled tetrasaccharide fraction (0.125 mg/kg) failed to modify the bronchoconstrictor responses to histamine (n = 3), carbachol (n = 3) and LTD_4 _(n = 3). The mean ± SE SR_L _values without and after pretreatment with inhaled tetrasaccharide were 509 ± 6% vs. 515 ± 5% (P = NS); 333 ± 21% vs. 368 ± 23% (P = NS); and 210 ± 35% vs. 222 ± 19% (P = NS) for histamine, carbachol and LTD_4_, respectively.

#### Histamine Release in BAL

Baseline concentration of histamine in BAL (n = 6) was 0.4 ± 0.2 nM and 0.5 ± 0.2 nM. Segmental antigen challenge caused a marked increase in BAL histamine concentration (8.5 ± 7.1 nM), which was not inhibited by pretreatment with inhaled tetrasaccharide (9.8 ± 8.1 nM).

#### Antifactor Xa-activity

In vitro the tetrasaccharide fraction had no anti-coagulant activity; the antifactor-Xa activity of tetrasaccharide fraction was 0.8 I.U.; while hexasaccharide and octasaccharide fractions had antifactor-Xa activity of 1.8 I.U. and 27 I.U., respectively.

### Murine Studies

**Figure 7:**
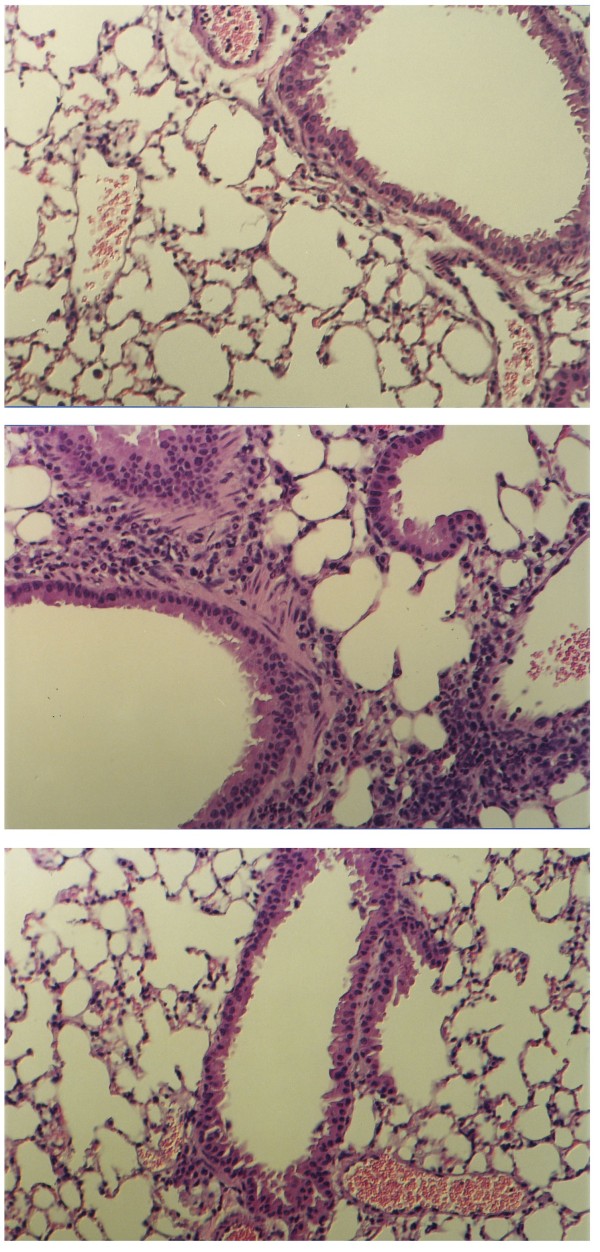
**Lung histology samples in ovalbumin immunized mice**. Top Panel: Control mice challenged with aerosolized saline; there is no evidence of peribronchial and perivascular inflammation. Middle Panel: Mice challenged with aerosolized ovalbumin; marked peribronchial and perivascular inflammation is seen with eosinophil infilteration. Bottom Panel: Ovalbumin challenged mice after pretreatment with nebulized heparin tetrasaccharide; reduced peribronchial and perivascular inflammation. Hematoxylin and eosin stain ( × 64 magnification).

In ovalbumin immunized mice, aerosolized ovalbumin (+controls) produced a marked perivascular and peribronchial inflammatory response and caused a significant increase in total cells and eosinophils in the BAL compared to immunized but saline challenged animals (-controls, Table 1). Pretreatment with aerosolized heparin-tetrasaccharide in immunized and ovalbumin challenged mice inhibited the inflammatory response in the peribronchial and perivascular regions (Figure 7). This histological finding was supported by the data in Table 1, which shows that heparin tetrasaccharide pretreatment reduced the total number of cells by 41%, the total number of eosinophils by 62% and the % eosinophils by 37% (all P < 0.05) when compared to the + controls. Table 1 also shows that there was a significant increase in the % macrophages and % neutrophils in the tetrasaccharide pretreated animals when compared to the + controls. However, calculation of the total neutrophil number shows the tetrasaccharide treated group (0.06 × 10^6 ^± 0.01) and the + control group (0.06 × 10^6 ^± 0.02) not to be different.

**Table 1:** Effect of heparin tetrasaccharide on ovalbumin (OVA) induced changes in inflammatory cell influx in BAL of mice

	Total	Total	%	%	%	%	%	%
	Cells	Eosinophils	Eosinophils	Epithelial	Macrophages	Lymphocytes	Neutrophils	Monocytes
**(-) Control** **(n = 4)**	0.46 × 10^6^	0.8 × 10^3^	0.2%	1.1%	94.2%	3.1%	0.2%	1.2%
	(0.04)	(0.3)	(0.1)	(0.6)	(2.6)	(2.0)	(0.1)	(0.4)
**(+) Control** **(n = 5)**	*1.02 × 10^6^	*0.45 × 10^6^	*44.3%	2.3%	*30.1%	*16.9%	*5.6%	0.8%
	(0.15)	(0.08)	(6.0)	(1.1)	(5.4)	(2.1)	(1.2)	(0.5)
**Tetrasaccharide** **(n = 4)**	†0.60 × 10^6^	†*0.17 × 10^6^	†*27.7%	3.7%	†* 42.8%	*12.4%	†*9.5%	1.1%
	(0.01)	(0.01)	(0.9)	(1.3)	(3.5)	(1.6)	(1.7)	(0.2)

Data are shown as mean (SE).

(-) Control (OVA sensitized/saline challenge)

(+) Control (OVA sensitized/OVA challenge)

Tetrasaccharide (Treatment group/OVA challenge)

* p < .05 from (-) control

† p < .05 from (+) control

### Chemical Structure Analysis

**Figure 8:**
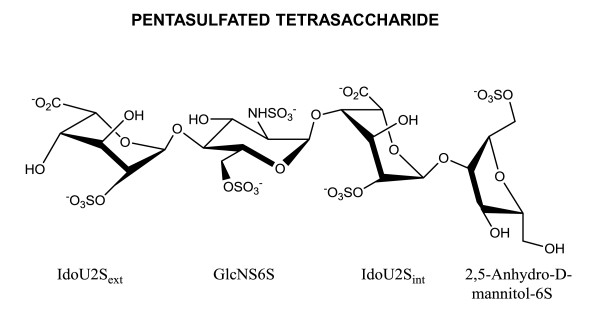
**Chemical structure of heparin-derived pentasulfated tetrasaccharide**.

Chemical structure analysis showed that the size-defined heparin-tetrasaccharide fraction is a mixture of tetrasaccharides. It predominantly consists of a pentasulfated tetrasaccharide (approximately 80%), along with other tetrasaccharides that have fewer sulfate moieties. The pentasulfated tetrasaccharide has a molecular weight of 1231 daltons. Its chemical structure consists of 2-*O*-sulfo- L-iduronic acid (1 → 4) 6-*O, N*-disulfo-D-glucosamine (1 → 4) 2-*O*-sulfo- L-iduronic acid (1 → 4) 6-*O*-sulfo-2,5-Anhydro-D-Mannitol [IdoU2S (1 → 4)GlcNS6S (1 → 4) IdoU2S (1 → 4) AMan-6S] (Figure 8).

## Discussion

Many biological actions of heparin, including the anti-allergic activity are molecular weight dependent [20-22]. Previous studies in sheep have demonstrated that unfractionated heparin inhibited the allergic airway responses only in "acute responders", while it was ineffective in "dual responders" [33]. In the "acute responder" sheep, the anti-allergic activity of fractionated heparin was molecular-weight dependent; and ultra low molecular weight heparin was found to be the most potent fraction [21,22]. We have also observed that the anti-allergic activity of fractionated heparin in the "dual responder" sheep is critically dependent on molecular weight. Only the ultra-low molecular weight heparin attenuated the antigen-induced EAR, LAR and AHR, while unfractionated heparin, medium molecular weight heparin and low molecular weight heparin were ineffective [23]. Thus, the ultra low molecular weight heparins (<2500 dalton) are qualitatively unique and inhibit allergic airway responses in both the acute and the dual responder sheep [21-23]. Although the anti-allergic activity of heparin is independent of its anticoagulant properties, the exact structural sequence and the chain-length possessing the anti-allergic activity is not known. The results of present study further extend our previous observations and demonstrate that: (a) the anti-allergic activity of heparin resides in a tetrasaccharide sequence, (b) the domains responsible for anticoagulant and anti-allergic activity of heparin are distinctly different, and (c) heparin tetrasaccharide possesses anti-inflammatory properties.

The heparin-derived oligosaccharide mixture used in the present study attenuated the allergic airway responses in both "acute" and "dual" responder sheep (figure 1 and 2). Its average molecular weight is 1930 daltons, and its anti-allergic activity is comparable to other oligosaccharides (mol wt 2200-2400 daltons) studied previously [21-23]. The size exclusion chromatographic analysis of heparin-derived oligosaccharide mixture showed that it is a heterogenous mixture, consisting of disaccharide, tetrasaccharide, hexasaccharide, octasaccharide and some decasaccharide fractions. Our data with different fractions revealed that the minimal oligosaccharide chain length possessing anti-allergic activity was a tetrasaccharide as disaccharide was ineffective. The tetrasaccharide, hexasaccharide and octasaccharide fractions inhibited the allergic airway responses in both "acute" and "dual" responders in a dose-dependent manner; however, the relative potency of each oligosaccharide increased with the smaller fractions. In the "acute responders", the octasaccharide fraction was two-fold more potent than the oligosaccharide mixture, while tetrasaccharide and hexasaccharide fractions were approximately eight-fold more potent. In the "dual responders", the anti-allergic activity of the octasaccharide fraction was comparable to the oligosaccharide mixture, while the hexasaccharide and the tetrasaccharide fractions were four-fold more potent. The results of this study also demonstrate that the tetrasaccharide fraction was also effective in inhibiting LAR and AHR, when administered after the antigen challenge. This action suggests possible anti-inflammatory activity as the LAR and AHR are pathophysiological indices of airway inflammation [27,28]. This conclusion is also supported by the mouse data (see below).

The heterogenous structure of glycosaminoglycan heparin is linked to its functional polydispersity. The biological activities of heparin are primarily mediated through its binding of various proteins and enzymes, but only the anti-thrombin III binding site involved in the anticoagulant action has been clearly elucidated as a distinct pentasaccharide sequence [25]. A hexasaccharide fraction has been suggested as the minimal chain length required to bind to fibroblast growth factor and transforming growth factor-β, as well as possessing antiproliferative activity on vascular smooth muscle cells [4,7,6]. The results of our studies demonstrate that heparin tetrasaccharide was the minimal chain length possessing the anti-allergic activity. Furthermore, the results of our studies are novel in that they demonstrate the unmasking of a biological action of heparin derived oligosaccharide (e.g. inhibition of LAR and AHR), which was not observed in unfractionated heparin or heparin fractions with molecular weight of >2500 daltons.

The basic structure of heparin consists of an alternating sequence of disaccharide units comprising of repeating 1→4 linked L-iduronic acid and D-glucosamine residues, which are variably sulfated [3,2]. However, the fine structure of heparin disaccharide units and its sulfation pattern varies with the method of depolymerization. The heparin oligosaccharides used in this study were produced by controlled nitrous acid depolymerization of porcine heparin, resulting in terminal glucosamine ring contraction to 2,5-anhydro-mannose and followed by its reduction to 2,5-anhydro-mannitol moiety. The tetrasaccharide fraction predominantly consists of a pentasulfated tetrasaccharide with a molecular weight of 1231 daltons. Its chemical structure consists of IdoU2S (1 → 4) GlcNS6S (1 → 4) IdoU2S (1 → 4) AMan-6S. Besides 2-0 and 6-0 sulfation, it has N-sulfation of the inner glucosamine ring, while the terminal glucosamine has ring contraction to 2,5-Anhydro-D-Mannitol-6S.

The anticoagulant action of heparin is predominantly mediated by binding of antithrombin III, which requires a pentasaccharide sequence with 3-0 sulfation of the inner glucosamine ring [25]. Previous studies in human subjects and sheep have suggested that the anti-allergic activity of inhaled heparin is independent of its anticoagulant properties, as APTT and antifactor-Xa activity were not prolonged [15,17,18,16]. This was also supported by the observations that non-anticoagulant fractions of heparin had potent anti-allergic activity [22,23,34]. The results of present study confirm this hypothesis. The heparin derived tetrasaccharide has an antifactor Xa activity of 0.8 IU; it also lacks critical 3-0 sulfation of the inner glucosamine ring, and its chain length is below the pentasaccharide sequence needed for anticoagulant activity. Collectively, these facts support the concept that the domains responsible for anti-coagulant and anti-allergic activity of heparin are distinctly different.

The mechanism of the anti-allergic activity of heparin derived oligosaccharides including the tetrasaccharide fraction is not known at present. Heparin derived tetrasaccharide failed to modify the bronchoconstrictor responses induced by the smooth muscle agonists, including histamine, carbachol or LTD_4_; thus excluding a direct effect on airway smooth muscle. The tetrasaccharide fraction had no effect on antigen-induced histamine release in BAL. It has been proposed that the anti-allergic activity of unfractionated heparin in acute responders may be mediated by inhibition of IP_3 _- dependent mast cell mediator release [35]. The inhibition of IP_3 _binding to its receptors by heparin is molecular weight dependent and the inhibitory activity decreases as the size of the heparin chain is reduced below 18 monosccharide units [36]. While fractions, containing 10-14 monosaccharide units, had substantially lower activity, the 8 monosaccharide fractions had none [36]. Thus, it is unlikely that the anti-allergic activity of heparin derived tetrasaccharide is related to inhibition of IP_3_-dependent mast cell mediator release. This is supported by our observation that the heparin derived tetrasaccharide failed to modulate antigen-induced histamine release in BAL fluid. The failure to modulate BAL histamine release but still have anti-allergic activity (blocking of EAR, LAR and AHR) is also consistent with our previous work which showed that structural domain of the heparin molecule responsible for this anti-allergic activity resides in the glycosaminoglycan chain length of <2500 daltons, whereas, the histamine-release inhibitory activity domain is located in the chain length of >2500 daltons [22,23]. As indicated above, all the fractions studied here were less < 2500 daltons.

The unfractionated heparin and low molecular weight heparins have been suggested to possess anti-inflammatory properties [37-40]. The attenuation of allergic airway responses without inhibition of histamine released in the BAL suggests that the in vivo activity of heparin tetrasaccharide may be mediated by a yet unknown anti-inflammatory mechanism. The inhibition of LAR and AHR by heparin tetrasaccharide, when administered after the antigen challenge supports this concept, as the immediate response to allergen is unimpaired, yet LAR and AHR, the primary physiological responses reflecting antigen-induced inflammation were blocked [41,28]. This concept is also supported by the observation that aerosolized heparin-tetrasaccharide caused a reduction in the allergen-induced eosinophil influx in the BAL fluid of ovalbumin sensitized mice and attenuated the peribronchial and perivascular inflammatory cell infiltration. The collective data supporting the anti-inflammatory actions of heparin tetrasaccharide seen here are consistent with reports that unfractionated heparin and heparin oligosaccharides, including tetrasaccharides, are effective inhibitors of L- P- selectins and demonstrate anti-inflammatory activity in vivo [42-44]. For example, intravenous heparin-tetrasaccharide was shown to reduce neutrophil influx in thioglycollate-induced peritoneal inflammation in mice [42], a process which has been shown to involve stimulation of both L- and P-selectin. Finally, the potential role of anti-selectin action of heparin tetrasaccharide in inhibiting allergic airway responses would also be consistent with our previous observation in sheep, showing attenuation of LAR and AHR by selectin inhibitors [45].

Although this study provides unique and novel data with regards to the anti-allergic and anti-inflammatory actions of oligosaccharides there are limitations. One short coming is that we are unclear as to the exact mechanism by which these oligosaccharides provide their protective effects. Having now described that the tetrasaccharide fraction is the minimal chain length to demonstrate these properties we can use this molecule to focus on the actual mechanism(s) involved in preventing these allergic responses. We should also point out that we did not measure the effects of these oligosaccharides on small airway function in this study. While our previous work has identified small airway dysfunction (i.e. decreased dynamic compliance) following allergen provocation [27], many of the studies designed to test the effects of agents on allergen-induced EAR, LAR and AHR have not involved these measures because SR_L _is more easily related to measures of airway function in patients in clinical trials (i.e. specific airway conductance or FEV_1_). Thus, while our data provides evidence that these oligosaccharides can modulate large airway effects associated with allergen challenge, we cannot categorically assume that small airway function was protected to a similar extent.

In conclusion, we have demonstrated for the first time that heparin tetrasaccharide is the minimal chain length that possesses anti-allergic and anti-inflammatory properties and these actions are distinct from any anti-coagulant properties. Our findings showing that both pre and post treatment modulate LAR and AHR, support the possibility that heparin oligosaccharides may interrupt multiple diverse cellular events involved in the complex cascade of allergic airway inflammation.

## Abbreviations

AHR: airway hyperresponsiveness; ABR: antigen-induced bronchoconstrictor response; BAL: Bronchoalveolar Lavage; EAR: early airway response; LAR: late airway response; EAR AUC_0-4h_: early response area under the curve; LAR AUC_4-8h_: late response area under the curve; PD_400_: cumulative provocating dose of carbachol (in breath units) that increased SR_L _to 400% above the baseline; SR_L_: specific lung resistance; SE: standard error; FEV_1_: forced expiratory volume in 1 second.

## Competing interests

T.A. is the inventor of US patents #5,980,865 and #6,193,957; assigned to OPKO HEALTH INC. He serves on the Scientific Advisory Board of OPKO HEALTH INC for which he receives stock options. None of the other authors have a financial relationship with a commercial entity that has an interest in the subject of this manuscript.

## Authors' contributions

TA and WMA designed the experimental studies, performed data analysis and wrote the manuscript. GS and IV isolated and characterized the different oligosaccharide fractions used in this study. All authors read and approved the final manuscript.
